# Sectoral analysis of the retinal nerve fiber layer thinning and its association with visual field loss in homonymous hemianopia caused by post-geniculate lesions using spectral-domain optical coherence tomography

**DOI:** 10.1007/s00417-015-3181-1

**Published:** 2015-10-07

**Authors:** Katsutoshi Goto, Atsushi Miki, Tsutomu Yamashita, Syunsuke Araki, Go Takizawa, Masaki Nakagawa, Yoshiaki Ieki, Junichi Kiryu

**Affiliations:** Department of Ophthalmology, Kawasaki Medical School, 577 Matsushima, Kurashiki city, Okayama Japan 701-0192; Doctoral Program in Sensory Science, Graduate School of Health Science and Technology, Kawasaki University of Medical Welfare, Kurashiki, Japan; Department of Sensory Science, Faculty of Health Science and Technology, Kawasaki University of Medical Welfare, Kurashiki, Japan

**Keywords:** Transsynaptic retrograde degeneration, Homonymous hemianopia, Post-geniculate visual pathway, Circumpapillary retinal nerve fiber layer, Spectral-domain optical coherence tomography

## Abstract

**Purpose:**

To report a sectoral analysis of circumpapillary retinal nerve fiber layer (cpRNFL) thinning and its association with visual field loss using spectral-domain optical coherence tomography (SD-OCT) in patients with homonymous hemianopia following acquired post-geniculate visual pathway damage.

**Patients and methods:**

Seven patients with homonymous hemianopia due to unilateral acquired post-geniculate visual pathway lesions were studied. The average duration from the onset of brain lesions to the initial visit was 49.8 months. Forty-nine normal control subjects without visual field defects, as confirmed using a Humphrey visual field analyzer, were also enrolled. Measurement of the cpRNFL thickness was performed at the initial visit and 24 months using SD-OCT (RTVue-100® OCT). The cpRNFL thickness was divided into eight sectors (superior temporal: ST, temporal upper: TU, temporal lower: TI, inferior temporal: IT, inferior nasal: IN, nasal lower: NL, nasal upper: NU, superior nasal: SN). The eye on the same side as the occipital lobe lesions was defined as the ipsilateral eye, and the eye on the opposite side was defined as the contralateral eye.

**Results:**

The average cpRNFL thickness in the homonymous hemianopic eyes was significantly reduced as compared with that seen in the normal controls, except for the ipsilateral eyes at the initial visit. Four of the eight sectors of the cpRNFL thickness in the homonymous hemianopic eyes were significantly reduced compared with that noted in the normal controls. In the ipsilateral eyes, the cpRNFL thickness in the ST, TU, TL, and IT sectors was significantly reduced at both the initial visit and 24 months. In the contralateral eyes, the cpRNFL thickness in the TU, TL, IT, and SN sectors was significantly reduced at both the initial visit and 24 months. The reduction of the quadrantic cpRNFL thickness significantly correlated with some of the visual field parameters, in accordance with the structure–function relationship. In the contralateral eyes, the T and I quadrant cpRNFL thickness correlated with the mean deviation and hemianopic field total deviation at 24 months. In the ipsilateral eyes, the S, T, and I quadrant cpRNFL thickness correlated with mean deviation. However, there were no correlations between the cpRNFL thickness and visual field parameters at the initial visit.

**Conclusions:**

A reduction of the cpRNFL thickness corresponding to the hemianopic visual field loss due to acquired post-geniculate visual pathway lesions was detected using SD-OCT, and the change was more evident at 24 months than at the initial visit. The latter finding suggests that this change is, at least partially, caused by transsynaptic retrograde degeneration.

## Introduction

The retinal nerve fiber layer is composed of the axons of retinal ganglion cells; most of these axons project to the lateral geniculate nucleus (LGN). The axons subsequently form synapses in the LGN and reach the primary visual cortex. It follows that patients who suffer damage to the optic nerve, optic chiasm, optic tract, or LGN will develop retrograde retinal ganglion cell atrophy. Therefore, optic chiasm and optic tract disorders cause characteristic hemianopic optic atrophy. Optic chiasm lesions lead to preferential atrophy of the temporal and nasal sectors of the optic disc due to damage of crossing fibers from nasal hemiretinae in both eyes, known as band atrophy (BA) or bow-tie-atrophy [1, 2]. Optic tract lesions also cause predominant atrophy of the superior and inferior sectors of optic disc due to damage of uncrossing fibers from temporal hemiretinae in the ipsilateral eye of the lesion, known as hour-glass atrophy, which leads to BA of the optic disc in the contralateral eye [3].

In animal experiments, atrophy of the LGN and retinal ganglion cells corresponding to the lesion has been reported after removal of the occipital lobe [4–6]. Using functional magnetic resonance imaging (MRI), decreased activation of the LGN on the affected side is observed in patients with post-geniculate lesions, suggesting retrograde degeneration and/or a functional decrease caused by decreased feedback from the ipsilateral visual cortex [7]. Transsynaptic retrograde degeneration (TRD) was considered in a patient with bilateral occipital lobe gun-related injury five and a half years earlier, although other explanations for the optic atrophy in that case are also possible [8]. However, it is generally believed that most post-geniculate visual pathway lesions do not induce TRD in humans. In human eyes, exceptional TRD occurs after congenital or long-standing occipital lobe lesions [9, 10]. Cowey [11] reported cpRNFL thinning in a patient with long-standing childhood-onset homonymous hemianopia using time-domain optical coherence tomography (TD-OCT). Metha and Plant [12] also showed thinning of the cpRNFL in long-standing/congenital occipital lobe lesions using TD-OCT. In a histological study of retinal ganglion cells, TRD was observed in a patient treated with the removal of the primary visual cortex 40 years ago [13]. In contrast, there is a report in which a patient with homonymous hemianopia showed normal optic discs 57 years after brain damage [14], suggesting that TRD does not necessarily occur in cases of long-standing acquired occipital lobe lesions.

Recent OCT studies have reported that the cpRNFL and retinal ganglion cell complex (GCC) thicknesses are reduced in homonymous hemianopia patients with acquired occipital lobe lesions. Jindahra et al. [15] reported that cpRNFL thinning is detected in both congenital and acquired homonymous hemianopia groups using TD-OCT. Furthermore, the authors demonstrated that thinning of the cpRNFL thickness in patients with homonymous hemianopia progresses within the first few months after brain damage due to stroke [16]. In our previous study, we reported that a reduction of the GCC thickness was detected in patients with posterior cerebral artery (PCA) infarction using spectral-domain OCT (SD-OCT) [17].

However, a few studies of relationship between visual field defects and the cpRNFL thickness have been reported in patients with homonymous hemianopia using SD-OCT. SD-OCT has a clear advantage in terms of providing high-speed scans with high resolution and more accurate segmentation of the retinal layer compared to TD-OCT, and is capable of evaluating more detailed pathologic changes [18, 19]. We performed a detailed analysis of the relationship between the visual field and the cpRNFL thickness in each eye using RTVue-100®. Therefore, the purpose of the present study was to analyze the sectoral cpRNFL thickness and to investigate its association with visual field parameters using SD-OCT in patients with homonymous hemianopia due to acquired post-geniculate visual pathway lesions.

## Materials and methods

Patients with homonymous hemianopia diagnosed using MRI due to unilateral occipital lobe lesions agreed to participate in this study at the Department of Ophthalmology at Kawasaki Medical School Hospital. Normal subjects recruited as an age-matched control group were also enrolled. The study protocol adhered to the tenets of the Declaration of Helsinki and was approved by the institutional review board of Kawasaki Medical School.

All patients underwent ocular examinations including measurements of best-corrected visual acuity, slit-lamp examinations, intraocular pressure assessments measured with Goldmann applanation tonometry, funduscopy, fundus photographs, visual field tests, and SD-OCT. Homonymous hemianopia patients underwent assessments with a Humphrey field analyzer® (Carl Zeiss Meditec., Dublin, CA, USA) using the central 30-2 Fast-pack program and Goldmann perimetry (Haag–Streit AG, Bern, Switzerland). Normal subjects underwent evaluations with a Humphrey field analyzer using the central 30-2 Swedish Interactive Threshold Algorithm (SITA) program.

Homonymous hemianopia on the Humphrey field analyzer was defined as at least four pairs along the vertical median with a difference in sensitivity of 2 dB or more and three pairs with a difference of 3 dB or more [20]. A normal visual field was defined as the absence of any clusters of at least three points with *P* < 5 %, one point with *P* < 0.5 % or 1 % on the pattern deviation probability plot, excluding the two points above and below the blind spot and within the normal limits on a glaucoma hemifield test with the 95 % confidence interval. Reliable visual field results obtained with the Humphrey field analyzer were defined as fixation loss of less than 20 % and false-positive and false-negative error of less than 20 %. Humphrey visual field parameters evaluated on the initial visit included the mean deviation (MD), pattern standard deviation (PSD) and hemianopic field total deviation (hemianopic TD:H-TD). The average H-TD values were obtained from 38 points in the hemianopic nasal hemifield and 34 points in the hemianopic temporal hemifield, excluding the two points immediately above and below the blind spot.

The inclusion criteria were as follows: best-corrected visual acuity 20/40 or better, range of spherical refractive power from −5.75 diopters (D) to +2.75D, cylinder refractive power within ±3.00D, intraocular pressure <22 mm Hg and no history of intraocular surgery, trauma, or retinal disease, including diabetic retinopathy, optic nerve disease, such as glaucoma, or any other diseases affecting the visual field. Patients with cataracts affecting the quality of the SD-OCT images were excluded.

## cpRNFL thickness measurements using SD-OCT

The SD-OCT examinations were performed using RTVue-100® (Optovue Inc., Fremont, CA, USA) at the initial visit and 24 months. The specifications for SD-OCT were such that the light source was a 840 nm superluminescent diode with a spectrum band width of 50 nm, axial resolution of 5.0 μm, A-scan/second of 26,000 and focus range of −15.00 D to +20.00 D. The RTVue-100® software program version 4.0 was used for the data analysis.

**Fig. 1:**
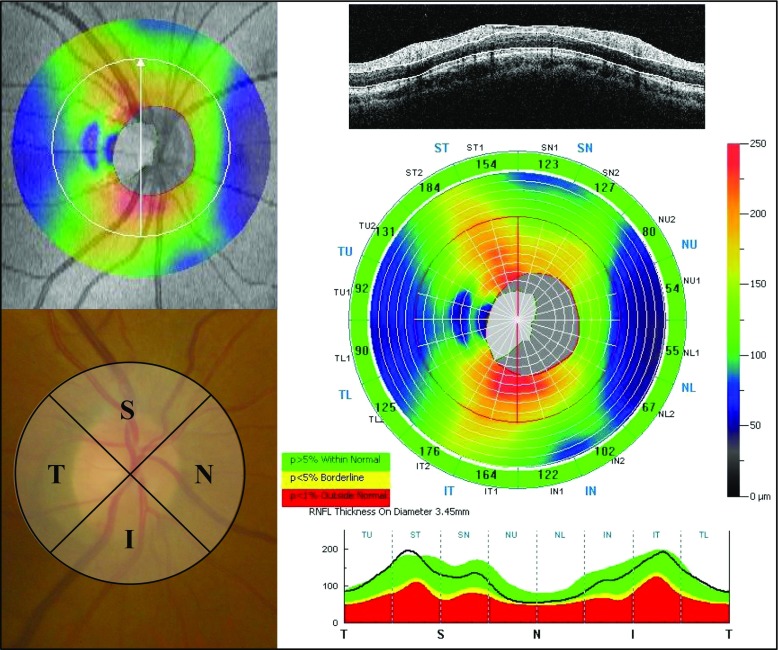
Four quadrants and eight sectors of the cpRNFL thickness using the ONH protocol. The ONH measurements were performed with 13 concentric circle ring scans and 12 radial line scans. The cpRNFL thicknesses in the four quadrants and eight sectors were defined as follows: superior (*S*), temporal (*T*), inferior (*I*), nasal (*N*), superior temporal (*ST*), temporal upper (*TU*), temporal lower (*TL*), inferior temporal (*IT*), inferior nasal (*IN*), nasal lower (*NL*), nasal upper (*NU*), and superior nasal (*SN*). *cpRNFL* circumpapillary retinal nerve fiber layer, *ONH* optic nerve head map

The optic nerve head map (ONH) protocol was used to obtain the cpRNFL thickness and optic disc parameters. This protocol is based on the three-dimensional baseline mode in which a 6 × 6 mm area scan is centered on the optic disc. The shape of the optic disc margin and anchoring point of the retinal pigment epithelium were defined according to the three-dimensional baseline mode automatically. Then, 13 concentric circle ring scans of 1.3 to 4.9 mm in diameter (425–965 A scans each) and 12 radial line scans of 3.4 mm (452 A scans each) were centered on the optic disc of a 4.9 mm area. The cpRNFL thickness on a circle 3.45 mm in diameter from the center of the optic disc was divided into 16 sectors. The 16 sectors were transformed as follows: four quadrants: superior (S), temporal (T), inferior (I), nasal (N), eight sectors: superior temporal (ST), temporal upper (TU), temporal lower (TL), inferior temporal (IT), inferior nasal (IN), nasal lower (NL), nasal upper (NU), superior nasal (SN), as shown in Fig. 1.

The eye on the same side of the occipital lobe lesion was defined as the ipsilateral eye (nasal hemianopia eyes) and the eye on the opposite side was defined as the contralateral eye (temporal hemianopia eyes). The cpRNFL measurements were performed several times, and the most reliable data were selected. Images with a signal strength index score of less than 45 were excluded. The visual field tests were performed within 3 months from the initial SD-OCT examination. The SD-OCT examinations were performed by experienced technicians (K.G., S.A.). The SD-OCT results were analyzed by neuro-ophthalmology specialists (A.M., T.Y.).

## Statistical analysis

The statistical analysis was performed using the Statistical Package for Social Science software package version 22.0 (SPSS, IBM, Tokyo, Japan). The Mann–Whitney *U* test and Fisher’s exact test were used to detect differences in the demographic characteristics of the normal control subjects and homonymous hemianopia patients. The average, quadrants, and eight sector cpRNFL thickness values in the ipsilateral eyes and contralateral eyes were compared with those of the normal control eyes using a one-way analysis of variance (one-way ANOVA). Dunnett’s post hoc multiple comparison was used if there was a significant difference in the one-way ANOVA. A second-order polynomial regression or a liner regression was used to evaluate the relationships between the cpRNFL thickness and visual field parameters. The Akaike information criterion (AIC) was used to determine an optimal model, which included liner and quadratic terms for cpRNFL thickness and visual field parameters. A statistically significant difference was defined as a *p* value of less than 5 %.

## Results

Seven patients with homonymous hemianopia due to acquired post-geniculate visual pathway lesions and 49 normal control subjects were included in this study. The clinical data of the patients are shown in Table 1. Of the seven patients with occipital lobe lesions, six patients had PCA infarction and one patient had intracerebral hemorrhage in the occipital and parietal lobes. There were no significant differences in age or refractive error between the homonymous hemianopic eyes and normal control eyes (Table 2). The average duration of the post-geniculate visual pathway lesions was 49.8 ± 70.5 months (range, 3.5 to 198.4 months). The Humphrey visual field parameters are shown in Table 3.

**Table 1:** Clinical data for patients with homonymous hemianopia

Case	Gender	Age (years)	Refractive error (spherical equivalent)	Hemianopic side	Visual field	Cause of visual field	Disease duration (months)
1	M	76	R) −0.75D, L) +0.50D	Right	Inferior quadrantanopia	infarction of left PCA territory	77.0
2	F	64	R) +1.00D, L) +0.75D	Right	Inferior quadrantanopia	infarction of left PCA territory	198.4
3	F	70	R) +0.25D, L) +1.00D	Left	Complete homonymous hemianopia	infarction of right PCA territory	22.8
4	F	39	R) −0.75D, L) −1.50D	Right	Incomplete homonymous hemianopia	infarction of left PCA territory	3.5
5	M	74	R) −1.25D, L) +1.25D	Left	Complete homonymous hemianopia	infarction of right PCA territory	36.2
6	F	70	R) +2.25D, L) +2.50D	Left	Incomplete homonymous hemianopia	right intracerebral hemorrhage in occipital and parietal lobe	6.0
7	M	67	R) −4.75D, L) −1.75D	Left	Complete homonymous hemianopia	infarction of right PCA territory	5.0

*M* male, *F* female, *D* diopter, *R* right, *L* left, *PCA* posterior cerebral artery

**Table 2:** Demographic characteristics of the normal control subjects and patients with homonymous hemianopia

	Homonymous hemianopia patients (*n* = 7)		Normal control subjects (*n* = 49)	
	Ipsilateral eyes	Contralateral eyes	Normal eyes	*P* values
Age (years)	65.7 ± 12.4		63.2 ± 7.9	0.192
Gender (M:F)	3 : 4		15 : 34	0.669
Refractive error (D)	−0.54 ± 2.25	0.36 ± 1.48	−0.34 ± 1.87	0.959 / 0.588

*M* male, *F* female, *D* diopter

**Table 3:** Visual field parameters using a Humphrey field analyzer in patients with homonymous hemianopia

	Visual field parameters (mean values)		
	MD (dB)	PSD (dB)	H-TD (dB)
Ipsilateral eyes	−16.17	14.45	−24.23
Contralateral eyes	−13.56	14.05	−25.32

*MD* mean deviation, *PSD* pattern standard deviation, *H-TD* hemianopic field total deviation, *dB* decibel

**Fig. 2:**
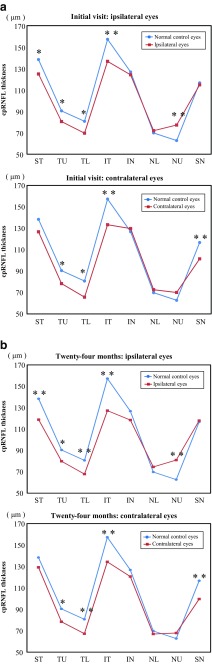
**a** Eight sectors of the cpRNFL thickness evaluated using SD-OCT at the initial visit. In the ipsilateral eyes, the cpRNFL thicknesses in the ST, TU, TL, and IT sectors were significantly reduced, although that in the NU sector was increased. In the contralateral eyes, the cpRNFL thicknesses in the TU, TL, IT, and SN sectors were significantly reduced. *SD-OCT*: spectral-domain optical coherence tomography * *P* < 0.05 **P < 0.01 between the groups. **b** Eight sectors of the cpRNFL thickness evaluated using SD-OCT at 24 months. In the ipsilateral eyes, the cpRNFL thicknesses in the ST, TU, TL, and IT sectors were significantly reduced, while that in the NU sector was increased. In the contralateral eyes, the cpRNFL thicknesses in the TU, TL, IT, and SN sectors were significantly reduced. *P < 0.05 **P < 0.01 between the groups

The average cpRNFL thicknesses at the initial visit and 24 months were 99.93 ± 15.53 and 98.20 ± 14.02 μm in the ipsilateral eyes and 97.20 ± 14.11 and 95.49 ± 13.46 μm in the contralateral eyes, respectively. On the other hand, the average cpRNFL thickness in the normal subjects was 107.02 ± 6.81 μm. There was a significant difference in the average cpRNFL thickness between the homonymous hemianopic eyes and the normal eyes, except for the ipsilateral eyes on the initial visit. In the quadrantic analysis, some of the cpRNFL thicknesses were observed to be significantly different between the normal control subjects and homonymous hemianopic patients. In the ipsilateral eyes, the cpRNFL thickness was significantly reduced in the T quadrant on the initial visit and in the T and I quadrants at 24 months, although there was an increase in the N quadrant at 24 months. In the contralateral eyes, the cpRNFL thickness was significantly reduced in the S and T quadrants on the initial visit and the S, T, and I quadrants at 24 months (Table 4). With regard to the eight-sector analysis, some of the cpRNFL thicknesses in the homonymous hemianopia patients were significantly reduced compared with that seen in the normal control subjects. In the ipsilateral eyes, the cpRNFL thicknesses in the ST, TU, TL, and IT sectors were significantly reduced at both the initial visit and 24 months, while an increased thickness was found in the NU sector. In the contralateral eyes, the cpRNFL thicknesses in the TU, TL, IT, and SN sectors were significantly reduced at both the initial visit and 24 months (Fig. 2a-b). Nevertheless, none of the patients were found to have optic atrophy on the fundus examinations, including the review of the fundus photographs.

**Table 4:** cpRNFL thicknesses obtained using SD-OCT in the normal eyes and patients with homonymous hemianopia

	cpRNFL thickness (μm)					
	At the initial visit		At 24 months			
	Ipsilateral eyes	Contralateral eyes	Ipsilateral eyes	Contralateral eyes	Normal control eyes	*P* values
Average	99.93 ± 15.53	97.20 ± 14.11*	98.20 ± 14.02*	95.49 ± 13.46**	107.02 ± 6.81	0.108 / 0.018 / 0.028 / 0.003
Quadrants						
Superior	119.79 ± 13.71	114.04 ± 11.48*	118.32 ± 17.23	114.36 ± 13.84*	127.48 ± 11.74	0.227 / 0.016 / 0.161 / 0.029
Temporal	75.00 ± 10.92*	71.96 ± 8.24**	73.86 ± 9.70**	72.79 ± 11.23**	85.54 ± 8.86	0.012 / 0.001 / 0.006 / 0.003
Inferior	130.36 ± 24.01	131.50 ± 22.48	122.82 ± 21.24**	127.43 ± 20.02*	141.98 ± 11.33	0.119 / 0.173 / 0.003 / 0.028
Nasal	74.57 ± 11.26	71.29 ± 15.72	77.79 ± 9.74**	67.39 ± 12.50	66.25 ± 7.86	0.072 / 0.359 / 0.004 / 0.937
Eight sectors						
ST	124.86 ± 16.61*	126.64 ± 11.28	118.79 ± 22.53**	129.14 ± 13.73	138.33 ± 12.57	0.024 / 0.057 / 0.002 / 0.206
TU	80.43 ± 16.01*	78.29 ± 10.91*	79.86 ± 15.28*	78.36 ± 18.13*	90.40 ± 9.57	0.044 / 0.012 / 0.049 / 0.022
TL	69.57 ± 9.59*	65.64 ± 8.91**	67.86 ± 7.60**	67.21 ± 11.81**	80.68 ± 9.88	0.013 / 0.001 / 0.004 / 0.003
IT	136.64 ± 30.20**	133.29 ± 17.00**	127.21 ± 24.44**	134.29 ± 18.58**	157.22 ± 12.24	0.003 / 0.001 / 0.001 / 0.001
IN	124.07 ± 24.52	129.71 ± 37.03	118.43 ± 24.90	120.57 ± 28.49	126.73 ± 15.41	0.931 / 0.914 / 0.456 / 0.645
NL	71.93 ± 12.52	72.57 ± 16.92	74.57 ± 10.43	66.93 ± 13.02	69.80 ± 8.42	0.839 / 0.744 / 0.363 / 0.687
NU	77.21 ± 12.78**	70.00 ± 20.09	81.00 ± 10.84**	67.86 ± 16.38	62.70 ± 7.84	0.002 / 0.160 / 0.001 / 0.322
SN	114.71 ± 13.34	101.43 ± 15.51**	117.86 ± 15.84	99.57 ± 17.76**	116.63 ± 13.32	0.925 / 0.015 / 0.971 / 0.008

Dunnett’s post hoc multiple comparison test : ipsilateral and contralateral eyes were compared with normal control eyes

*cpRNFL* circumpapillary retinal nerve fiber layer, *SD-OCT* spectral-domain optical coherence tomography, *ST* superior temporal, *TL* temporal lower, *IT* inferior temporal, *IN* inferior nasal, *NL* nasal lower, *NU* nasal upper, *SN* superior nasal

* *p* < 0.05 **p < 0.01

**Fig. 3:**
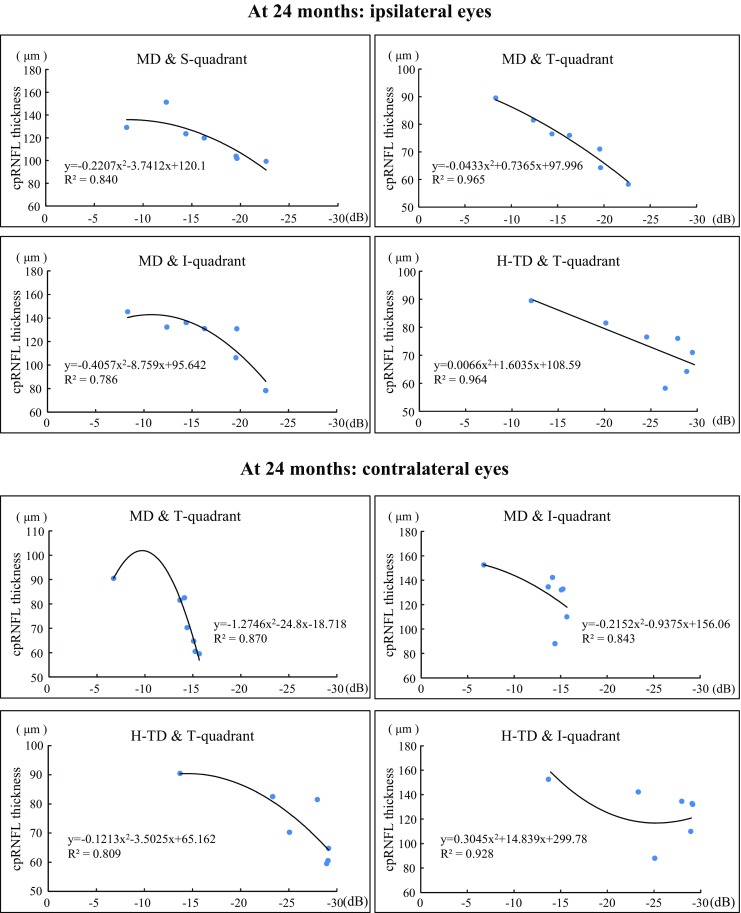
Scatter plot of the cpRNFL thickness against the visual field parameters. In the ipsilateral eyes, the values in the S, T, and I quadrants at 24 months correlated with MD. In the contralateral eyes, the T and I quadrant values at 24 months correlated with both MD and H-TD. *S* superior, *T* temporal, *I* inferior, *N* nasal

With regard to the relationship between visual field defects and the cpRNFL thickness, some of the cpRNFL thicknesses significantly correlated with visual field parameters at 24 months in the ipsilateral eyes and contralateral eyes, although there was no correlation at the initial visit. In the ipsilateral eyes, the values in the S, T, and I quadrants correlated with MD, and the T quadrant correlated with H-TD. On the other hand, in the contralateral eyes, the values in the T and I quadrants correlated with both MD and H-TD (Fig. 3 and Table 5).

**Table 5:** Coefficient of determination with between the cpRNFL thickness and visual field parameters in patients with homonymous hemianopia

	At the initial visit						At 24 months					
	Ipsilateral eyes			Contralateral eyes			Ipsilateral eyes			Contralateral eyes		
	Coefficient of determinaton R^2^											
	MD	PSD	H-TD	MD	PSD	H-TD	MD	PSD	H-TD	MD	PSD	H-TD
cpRNFL												
Quadrants												
Superior	0.643	0.477	0.295	0.428	0.428	0.096	0.840**	0.532	0.471	0.080	0.567	0.011
Temporal	0.740	0.471	0.690	0.103	0.103	0.014	0.965**	0.650	0.964**	0.870*	0.179	0.809*
Inferior	0.701	0.486	0.548	0.738	0.738	0.730	0.786**	0.457	0.724	0.843*	0.418	0.928**
Nasal	0.636	0.414	0.410	0.624	0.624	0.562	0.611	0.722	0.206	0.206	0.329	0.239

A second-order polynomial regression

*cpRNFL* circumpapillary retinal nerve fiber layer, *MD* mean deviation, *PSD* pattern standard deviation, *H-TD* hemianopic field total deviation

**p < 0.01, *p < 0.05

## Case reports

### Case 4

**Fig. 4:**
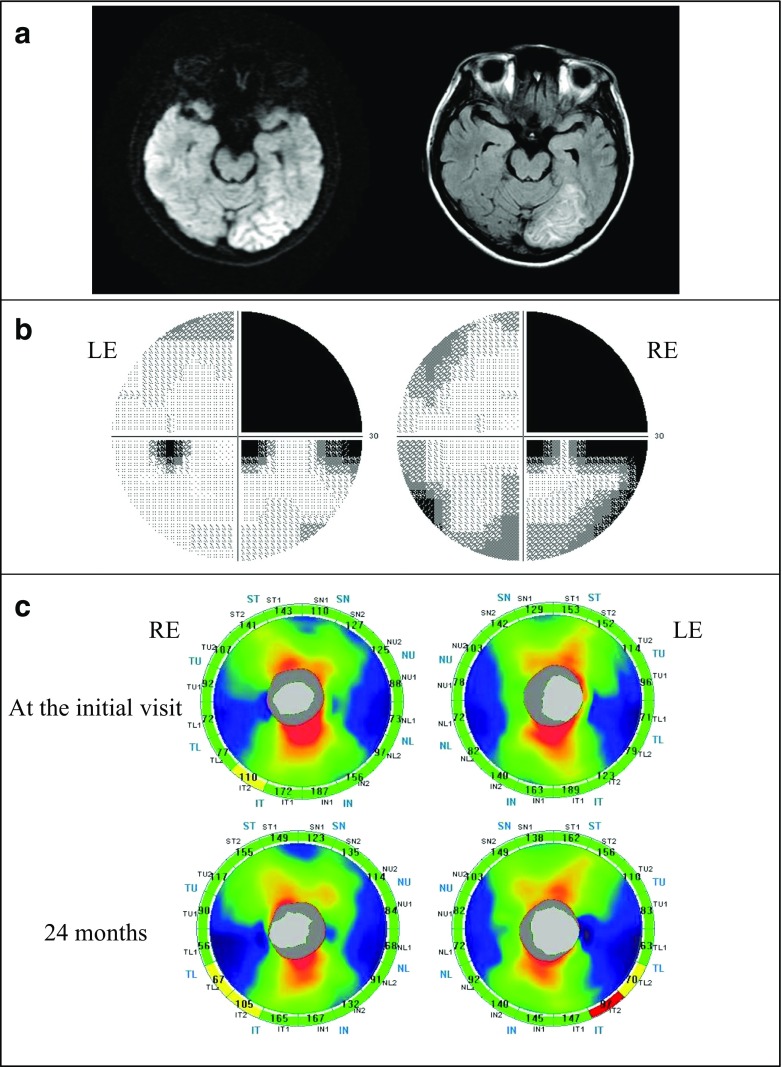
Patient with right homonymous hemianopia (case 4). **a** A 39-year-old woman with an infarction in the left posterior cerebral artery territory. *Left*: a diffusion weighted image (DWI), *Right* a fluid attenuated inversion recovery (FLAIR) image. DWI and FLAIR images showed an ischemic stroke on the left occipital lobe which was represented as a high-intensity lesion 3.5 months after the onset. **b** Humphrey visual fields showed right homonymous hemianopia denser superiorly. *RE* right eye, *LE* left eye. **c** A Significance map of the cpRNFL thickness. *Top*: at the initial visit, *Bottom*: 24 months after the onset. *RE* right eye, *LE* left eye. At the initial visit, cpRNFL thinning was observed in the inferior temporal sector of the RE. There was no significant change in the LE. After 24 months, the thinning of RE was progressive and thinning sectors of LE corresponded to the visual field defect

A 39-year-old woman had been treated for an infarction in the left posterior cerebral artery region. The best-corrected visual acuity was 1.5 in both eyes. No abnormality was observed in the intraocular pressure, anterior segments, optic media, or ocular fundus. The MRI images showed an ischemic stroke on the left occipital lobe which was represented as a high-intensity lesion 3.5 months after the onset (Fig. 4a). A Humphrey field analyzer showed right homonymous hemianopia denser superiorly (Fig. 4b). At the initial visit, mild cpRNFL thinning was observed only in the inferior temporal sector of the right eye (Fig. 4c, Top). After 24 months, thinning of the right eye was found in two sectors. In addition, a newly appeared sector with cpRNFL thinning in the left eye was found. These sectors were located inferiorly in accordance with the visual field defects denser superiorly (Fig. 4c, Bottom).

### Case 7

**Fig. 5:**
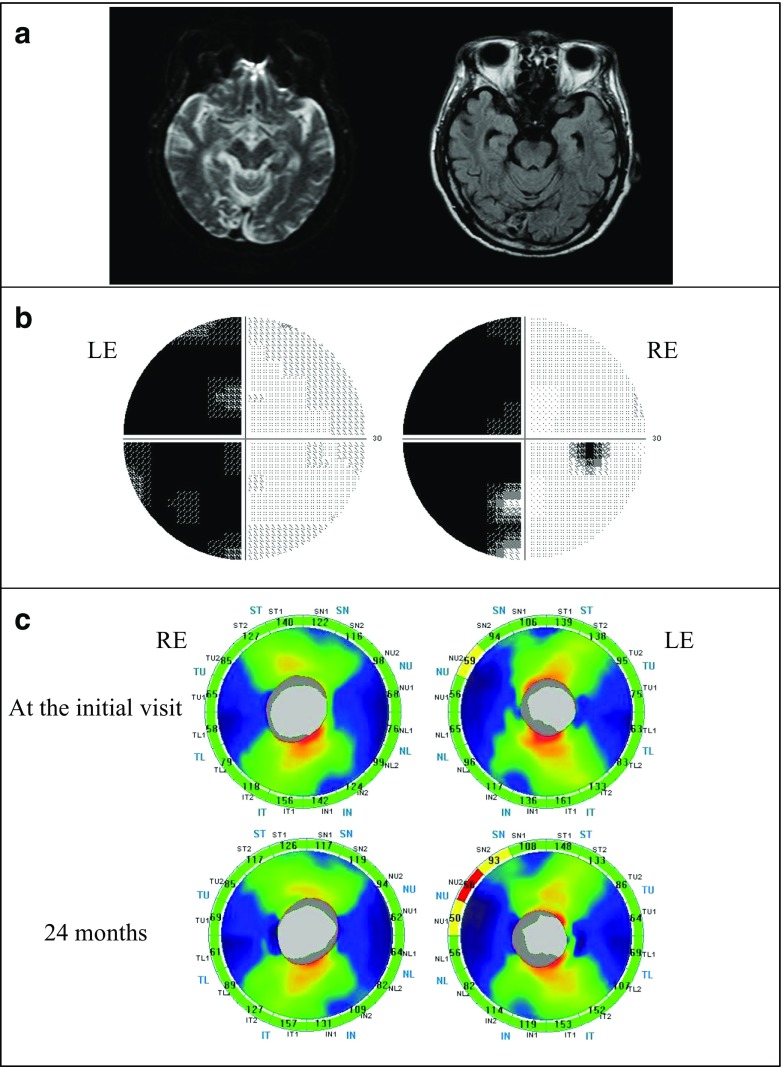
Patient with left homonymous hemianopia (case 7). **a** A 67-year-old man with an infarction in the right posterior cerebral artery territory. *Left*: DWI, *Right*: FLAIR. DWI and FLAIR images showed an ischemic stroke on the right occipital lobe, which was represented as a hyper- and mixed-intensity lesion 5 months after the onset. **b** Humphrey visual fields showed left homonymous hemianopia with macular splitting. **c** A Significance map of the cpRNFL thickness. *Top*: at the initial visit, *Bottom*: 24 months after the onset. There was no thinning area in the RE at both visits. At the initial visit, cpRNFL thinning was observed corresponding to the visual field defect in the superior nasal sector of the LE, and the thinning showed a progression at 24 months

A 67-year-old man had been treated for a right posterior cerebral artery infarction. The best-corrected visual acuity was 1.5 in both eyes. There was no abnormality in the intraocular pressure, anterior segments, optic media, or ocular fundus. MRI showed an ischemic stroke on the right occipital lobe which was represented as a hyper- and mixed-intensity lesion 5 months after the onset (Fig. 5a). A Humphrey field analyzer showed left homonymous hemianopia with macular splitting (Fig. 5b). At the initial visit, a sector with cpRNFL thinning was found at the superior nasal area in the left eye, although no significantly thinned area was observed in the right eye (Fig. 5c, Top). After 24 months, thinning in the left eye progressed and spread to other sectors. The nasal areas of significant thinning in the left eye corresponded to the temporal hemianopic visual field defect (Fig. 5c, Bottom).

## Discussion

In this report, we investigated the time-course of the cpRNFL thickness using SD-OCT in patients with homonymous hemianopia due to post-geniculate lesions. The average cpRNFL thickness was significantly reduced in the patients with homonymous hemianopia compared with that seen in the normal control subjects, except for the ipsilateral eyes at the initial visit. Moreover, the cpRNFL thickness in some of the sectors or quadrants showed a significant decrease in the patient group. The duration between the onset of brain lesions and the examination in most of our patients was relatively short. These results demonstrate that thinning of the cpRNFL may be detected early after the onset of acquired brain lesions.

With regard to thinning of the cpRNFL in patients with homonymous hemianopia, there are a small number of reports using OCT [15, 16, 21]. Jindahra et al. [15] reported that the cpRNFL thickness determined using TD-OCT was significantly reduced in 19 patients with acquired homonymous hemianopia compared with normal controls. In particular, thinning of the cpRNFL was pronounced in the S, T, and I quadrants in the ipsilateral eyes and the T, S, and N quadrants in the contralateral eyes. In a subsequent study, Jindahra et al. [16] showed that that the cpRNFL thicknesses obtained using TD-OCT in 38 patients with acquired homonymous hemianopia rapidly reduced within the first few years and then decreased slowly thereafter (0.4 μm/year), while the degree of change in the cpRNFL thickness was 9.08 μm/log year. In terms of the time course of the cpRNFL thickness in their seven patients, a linear decrease 2 years after the onset of stroke was observed. Park et al. [21] reported that the cpRNFL thickness evaluated using Cirrus HD-OCT was reduced significantly in both eyes of 46 patients with cerebral infarction compared to normal control eyes. Notably, the cpRNFL thickness was reduced significantly in the S, I, and T quadrants in the ipsilateral eyes and the S, I, and N quadrants in the contralateral eyes.

In the present study, thinning of the cpRNFL in the ipsilateral eyes was observed in the ST, TU, TL, and IT sectors, and the results are consistent with those of other previous reports [15, 21]. Non-crossing fibers from temporal hemiretina mainly enter the superior and inferior sectors, including the temporal sector of the optic disc. Therefore, thinning of the temporal sectors in the ipsilateral eyes is associated with the anatomical structure of non-crossing fibers.

In the present study, thinning of the cpRNFL in the contralateral eyes was found in the TU, TL, IT, and SN sectors. Crossing fibers from the nasal hemiretina are mainly incident on the optic disc in the temporal and nasal sectors. However, no thinning was detected in the NU, NL, and IN sectors in the contralateral eyes in this study, and the values in the temporal sectors were predominantly reduced. This finding is not in agreement with that of other previous reports [15, 21], in that the nasal quadrant of cpRNFL was reduced significantly in the contralateral eyes. A possible explanation for the discrepancy is that we studied a fairly homogeneous group of patients, while the previous studies included heterogeneous patients. In addition, Ueda et al. [22] reported that cpRNFL originating from the nasal hemiretina predominantly enters into the optic disc at the 1 o’clock and 5 o’clock angles. They also showed that the sector with the greatest reduction in band atrophy eyes compared with normal eyes was at 1 o’clock. This finding is in accordance with ours that the SN sector in the contralateral eyes was significantly reduced in the present study. Another potential reason for our finding could be that the cpRNFL of the nasal sector corresponds to the presence of crossing fibers from ganglion cells of the peripheral retina. On the other hand, the cpRNFL in the temporal sectors of the optic disc corresponds to the existence of crossing fibers from the ganglion cells of the central retina, including the papillomacular bundle. In other words, the reduction in the cpRNFL in the temporal quadrant in the contralateral eyes is considered to reflect the prominent distribution of retinal ganglion cells in the central area. Weller and Kaas [23] reported that nearly 80 % of retinal ganglion cells were lost in macaque monkeys with striate cortex lesions in their experiment, and that the degeneration was pronounced in the ganglion cells projecting to the parvocellular layers of the lateral geniculate nucleus. Because the ganglion cells of the parvocellular layers mainly exist in the central retina, our findings also suggest that ganglion cell atrophy due to post-geniculate visual pathway lesions with homonymous hemianopia is relatively confined to the central retina. Therefore, we hypothesize that peripheral retinal ganglion cells are not significantly affected in this situation, and, as a result, the nasal quadrant of cpRNFL in the contralateral eyes is not reduced. Thus, our results in the contralateral eyes are quite reasonable if we consider the incident position of crossing fibers from the nasal hemiretina and the preferential loss of central retinal ganglion cells.

Our results may have reflected more detailed pathological changes than those of Jindahra et al. [15], as SD-OCT has a clear advantage in terms of providing high-speed, high-resolution accurate segmentation of cpRNFL compared with TD-OCT. Measurements of the cpRNFL thickness using SD-OCT have been shown to have high repeatability and excellent reproducibility in both normal and glaucomatous eyes [24, 25]. SD-OCT is also superior to TD-OCT in the detection of cpRNFL progression in glaucoma patients and in the normative classification of average cpRNFL thickness [26, 27]. Therefore, we consider that SD-OCT is suitable for detecting and monitoring cpRNFL atrophy in patients with homonymous hemianopia due to post-geniculate lesions.

In the present study, the reduction of the cpRNFL thickness in the patients with acquired post-geniculate lesions was found. Previous studies of cpRNFL using SD-OCT in humans with occipital lobe lesions have attributed thinning of the cpRNFL to TRD [15, 16, 21]. Since TRD should be progressive rather than abrupt, our results also suggest that reduction of the cpRNFL thickness in patients with acquired post-geniculate lesions is caused by TRD of retinal ganglion cells, in agreement with other previous reports. Therefore, measuring the cpRNFL thickness is useful for evaluating retinal ganglion cell atrophy in patients with homonymous hemianopia due to post-geniculate visual pathway lesions. Another explanation for our findings may be the direct effects of ischemic damage in the anterior visual pathway, including the LGN. In the present study, our patients seemed to have a normal LGN on MRI. However, there is no clear evidence that the LGN was not affected by infarction. In fact, our finding that the cpRNFL thicknesses were already reduced at the initial examination rather supports the notion that the changes were caused by direct ischemic events.

With regard to investigations of other SD-OCT parameters, we previously reported that thinning of the GCC corresponding to the side of hemianopia was observed in three patients with PCA infarction [17]. In these patients, there were no clear abnormalities of the optic tract on MRI and/or optic nerve atrophy or retinal nerve fiber layer defects on fundus examinations. Keller et al. [28] reported that significant thinning of the macular ganglion cell layer in the sector corresponding to occipital cortex lesions was observed in eight patients with retrogeniculate lesions. Moreover, Tanito et al. [29] reported that the inner retinal thickness was reduced corresponding to infarction in a patient with a 10-year history of hemianopia due to unilateral posterior cerebral artery infarction. These results indicate that acquired post-geniculate visual pathway lesions associated with homonymous hemianopia lead to macular retinal ganglion cell atrophy.

Regarding the relationships between the visual field parameters and the cpRNFL thickness, we found that MD correlated with the cpRNFL thickness in the S, T, and I quadrants at 24 months. Also, H-TD correlated with the T quadrant at 24 months in the ipsilateral eyes. In the contralateral eyes, the T and I quadrant values correlated with MD and the H-TD at 24 months. These results are well in accordance with the structural changes in which the cpRNFL thickness was reduced predominantly in the S, T, and I sectors in the ipsilateral eyes and the T sector in the contralateral eyes. Our results indicate that the reduction of the cpRNFL thickness due to hemianopic visual field defects corresponds to the values in the S, T, and I quadrants in the ipsilateral eye and the T quadrant in the contralateral eye. Jindahra et al. [30] reported that the cpRNFL thickness correlates well with the MD in patients with homonymous hemianopic or quadrantanopic field loss. Our findings also suggest that the change in the cpRNFL thickness corresponds to the visual field sensitivity in each eye. In contrast, Keller et al. [28] reported that there was a significantly stronger correlation between the pattern deviation of visual field parameters and the corresponding GCL thickness rather than the cpRNFL thickness. They considered that it is more difficult to make a correlation of the visual field with the cpRNFL thickness than with the GCL thickness because the anatomical distribution of cpRNFL is more complex than that of GCL. We are planning to investigate the relationship between the macular inner retinal layer thickness and visual field defects in order to elucidate the effects on retinal ganglion cells in cases of post-geniculate visual pathway lesions.

There are several limitations associated with the present study. First, this study included a small number of patients. Additionally, the size and/or volume of the brain lesions and the time that had elapsed after the onset of the lesions were quite variable among the patients; however, an analysis of sub-groups was not feasible. Previous studies have reported that the size of striate cortex lesions corresponds to the extent of TRD of retinal ganglion cells [31, 32]. Further detailed research is necessary to elucidate the relationship between the size and/or location of brain lesions and damage to retinal ganglion cells. Hence, we plan to investigate the relationship between the infarcted region on MRI and SD-OCT parameters, including the macular inner retinal layer, in more detail.

In conclusion, we demonstrated thinning of the cpRNFL in patients with homonymous hemianopia due to unilateral acquired post-geniculate lesions. The thinning was detected early after onset, suggesting that the changes were caused by TRD. Moreover, the pattern of cpRNFL thinning correlated with hemianopic visual field defects in each eye. Measuring the cpRNFL thickness is useful for evaluating retinal ganglion cell atrophy due to occipital lobe lesions, as even experienced specialists cannot detect such atrophy using detailed fundus examinations. SD-OCT may also be used an index for quantifying post-geniculate visual pathway lesions in patients with dementia or higher brain dysfunction in whom subjective examinations, including visual field tests, are impossible.
